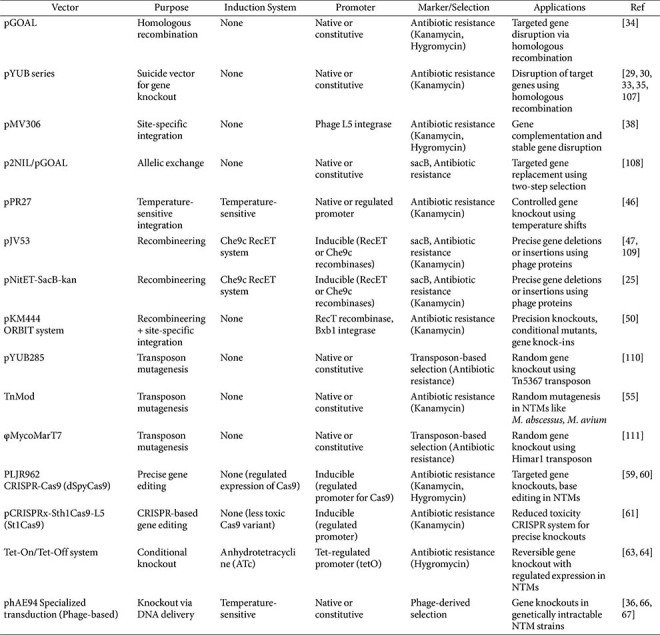# Advances and Challenges in Mycobacterial Genetic Engineering: Techniques for Knockout, Knockdown and Overexpression

**DOI:** 10.4014/jmb.2507.07051

**Published:** 2025-11-27

**Authors:** Thi Oanh Dao, Hyun-Eui Park, Jun Ho Lee, Kyu-Min Kim, Minh Phuong Trinh, Hyung-Lyun Kang, Han Sang Yoo, Min-Kyoung Shin

**Affiliations:** 1 Department of Microbiology and Convergence Medical Science, College of Medicine, Gyeongsang National University, Jinju 52727, Republic of Korea; 2 Department of Microbiology, Yonsei University Wonju College of Medicine, Wonju 26493, Republic of Korea; 3 Department of Global Medical Science, Yonsei University Wonju College of Medicine, Wonju 26493, Republic of Korea; 4 Department of Infectious Disease, Research Institute for Veterinary Science, BK21 FOUR Future Veterinary Medicine Leading Education and Research Center, College of Veterinary Medicine, Seoul National University, Seoul 08826, Republic of Korea

**Keywords:** Mycobacteria, mycobacterial genetic engineering, gene knockout, gene knockdown, overexpression

## Abstract

Genetic engineering of mycobacteria is challenging due to their hydrophobic cell wall structure and slow growth rates. Despite these obstacles, significant progress has been made to develop genetic engineering tools to study gene function and pathogenesis in these organisms. This review comprehensively explores the current methodologies employed in the genetic modification of mycobacteria, focusing on gene knockout, knockdown, and overexpression systems. Techniques covered include homologous recombination, recombineering, transposon mutagenesis, CRISPR-Cas systems, conditional expression strategies, and phage-mediated gene delivery. The mechanism, advantages, and limitations of those methods are critically analyzed, with particular emphasis on the adaptability of these tools to various mycobacterial species. By providing a detailed comparative analysis of available genetic tools, this review is a practical guide for researchers aiming to develop targeted and efficient genetic modifications in *Mycobacterium* species, accelerating discoveries in pathogenesis, drug resistance, and vaccine development.

## Introduction

The genus *Mycobacterium* comprises diverse species characterized by a complex, waxy cell wall that, along with other intrinsic mechanisms, makes them unusually resistant to antimicrobial agents and environmental stresses [1, 2]. Mycobacteria include important human and animal pathogens such as *Mycobacterium tuberculosis*, *Mycobacterium leprae*, *Mycobacterium bovis*, and *Mycobacterium paratuberculosis* [3-5]. Some mycobacterial species are opportunistic pathogens, while others are widely distributed in natural and human environments, including water, soil, dust, showerhead, and plumbing [6, 7].

Mycobacteria are responsible for a wide range of diseases in animals, impacting both domestic and wild species. Among these, *M. bovis* stands out as the causative agent of bovine tuberculosis, a highly contagious disease that can spread to humans and other animals [5, 8]. Another significant pathogen, *Mycobacterium avium*, primarily causes avian tuberculosis in birds but can also pose a threat to mammals [9]. Additionally, a subspecies of *M. avium*, known as *M. avium* subspecies *paratuberculosis* (MAP), is the etiological agent of Johne's disease, a chronic granulomatous enteritis that affects ruminants such as cattle, sheep, and goats [10]. These diverse mycobacterial infections underscore the broad host range and significant impact of this genus on animal health across species.

Non-tuberculous mycobacteria (NTM) have emerged as a significant global health concern due to their rising prevalence, challenging treatment regimens, and increasing drug resistance in both veterinary and human medicine [11-13]. Unlike *M. tuberculosis*, NTM are natural inhabitants of the environment and thereby exposed to humans through the soil and water [14, 15]. NTM infection threatens the susceptible population, such as older adults, immunocompromised individuals, and structural lung disease patients. The treatment of NTM infection is very challenging due to the emergence of antibiotic resistance and the side effects associated with long-term treatment regimens [16]. This growing global burden of NTM infections underscores the need for advanced research tools, and the study of NTMs can serve as a model for other mycobacterial diseases, providing insights that can be applied across a range of pathogens, including the including *M. tuberculosis* [11].

Given these challenges, the engineering of mycobacteria is of vital importance. By manipulating the genetics and physiology of these organisms, researchers can gain a deeper understanding of their pathogenic mechanisms, develop novel therapeutic strategies, and enhance diagnostic capabilities. However, engineering mycobacteria is far from straightforward. The unique biology of mycobacteria is defined by a highly complex and impermeable cell wall, composed of mycolic acids that form a dense, waxy outer layer, complex glycolipids that reinforce the barrier, and arabinogalactan, a polysaccharide linking peptidoglycan to the lipid layer [17]. This multilayered structure acts as a formidable defense against antibiotics, disinfectants, and genetic manipulation [18-20], and also makes it exceedingly difficult to introduce foreign DNA—a critical step in genetic engineering.

Moreover, mycobacteria have an inherently slow growth rate, with doubling times ranging from 12 to 24 h or even longer, which significantly slows down experimental progress [21]. Their genetic intractability adds another layer of complexity; mycobacteria are notoriously difficult to manipulate genetically due to challenges in DNA uptake, limited availability of selection markers, and complex gene regulation systems [22, 23]. Recombination and genetic stability present additional hurdles with frequent issues related to homologous recombination and plasmid instability that can undermine experimental results [24].

Despite these inherent difficulties, a variety of engineering methods and systems have been developed to manipulate mycobacteria, each with its own set of advantages and limitations[25, 26] (Table 1). Techniques such as CRISPR interference, transposon mutagenesis, and recombineering have expanded the toolbox available to researchers, but selecting the most suitable approach remains a critical decision that depends heavily on the specific objectives of the experiment. This review aims to guide researchers through the selection process by organizing and categorizing engineering methods based on experimental purposes. By providing a structured overview of the available tools, this work seeks to offer a practical guide for mycobacteria engineering, facilitating the design of more effective research strategies and accelerating the pace of discovery in the field of mycobacterial biology.

## Gene Knockout Techniques in Mycobacteria

Gene knockout methods are techniques used to inactivate or "knock out" a specific gene in an organism, allowing researchers to study the gene's function by observing the effects of its absence. The development of gene knockout techniques in NTM has been a critical component of mycobacterial research, helping to uncover the genetic basis of pathogenesis, drug resistance, and host-pathogen interactions. Over the decades, advancements in genetic tools have transformed how researchers approach NTM, enabling more sophisticated and targeted genetic manipulations.

### Homologous Recombination: Early Approaches and Challenges

The first gene knockout attempts relied on basic homologous recombination techniques, which work like a DNA repair mechanism that uses a DNA construct containing homologous sequences in the genome to copy damaged genetic information. The process begins with a double-strand break or single-strand gap in one of the DNA molecules due to DNA damage or errors during replication or intentional cleavage by an enzyme [27]. The enzyme RecBCD processes the broken DNA ends, trimming the 5' strands to generate single-stranded 3' overhangs. These overhangs search for a matching DNA sequence in a homologous DNA molecule. Upon finding a match, the single-stranded DNA invades the homologous molecule, forming a D-loop where pairing occurs. DNA synthesis repairs the gap, completing the recombination process [28]. The single-stranded DNA displaces one strand of the intact double-stranded DNA and pairs with its complementary sequence, forming a D-loop (displacement loop) followed by DNA synthesis, cutting and separating the Holliday junction, completing the recombination process.

**Fig. 1:**
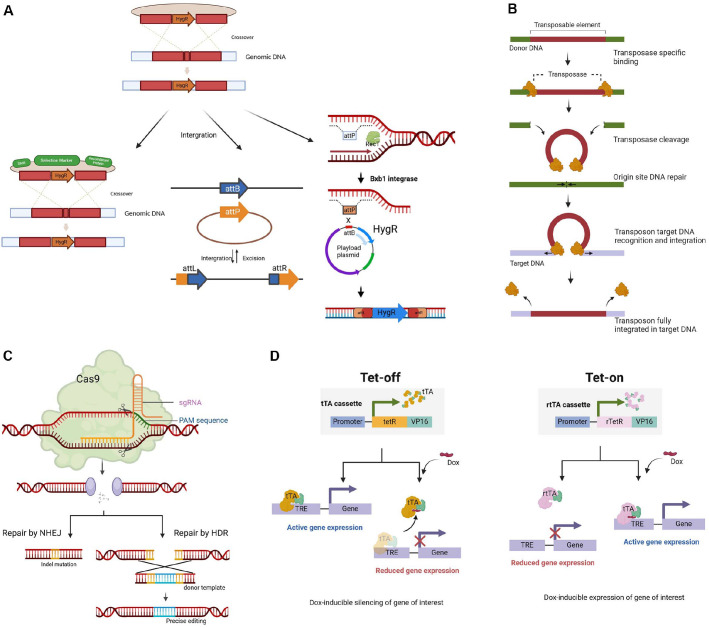
**(A) Homologous recombination** used for genetic manipulation in Mycobacteria including specialized vectors, such as those incorporating counter-selectable markers (*e.g.*, *sacB*), integrase system with site-specific integration at the attB site and the ORBIT system combines recombineering (RecT recombinase of phage Che9c) and site-specific integration (Bxb1) to achieve precise genetic modifications. **(B) Transposon mutagenesis.** The process begins with the donor DNA containing a transposable element, which binds to the transposase enzyme. Following transposase-specific binding, the enzyme cleaves the donor DNA, leading to the repair of the origin site DNA. Subsequently, the transposon recognizes and integrates into the target DNA. The final step shows the transposon fully integrated into the target DNA, resulting in a mutation that can disrupt gene function and facilitate the study of genetic pathways. **(C) CRISPR-Cas9** system utilizes a guide RNA (gRNA) to direct the Cas9 endonuclease to specific genomic loci, inducing double-strand breaks (DSBs). Once a DSB occurs, the cell can repair the breakthrough two primary mechanisms: Non-Homologous End Joining (NHEJ) and Homology-Directed Repair (HDR). NHEJ is an error-prone repair process that directly ligates the broken DNA ends, often resulting in insertions or deletions (indels) that can disrupt the target gene, effectively knocking it out. HDR, on the other hand, utilizes a homologous donor template to precisely repair the break, allowing for the introduction of specific mutations or the insertion of new sequences at the target site. **(D) Tet-On/Tet-Off system** utilizes the tet repressor (TetR) or tetracycline-controlled transactivator (tTA) to control transcription. In the Tet-on system, the addition of tetracycline induces gene expression by releasing TetR from the promoter, while in the Tet Off system, tetracycline prevents transcription by maintaining TetR's binding.

In the initial attempts, researchers attempted to manipulate genes through natural homologous recombination. However, the process was highly inefficient because of poor recombination frequencies and the difficulty of introducing DNA into the cells. The recombination machinery of the cell recognizes these homologous sequences and facilitates the exchange between the chromosomal DNA and the introduced construct, carrying DNA fragments with homologous regions flanking a selectable marker (*e.g.*, antibiotic resistance genes), leading to the replacement or disruption of the target gene (Fig. 1A). Significant progress was made in the late 1990s and early 2000s with the development of shuttle plasmids, which are capable of replicating in both *E. coli* and *Mycobacterium* species and facilitated the introduction of foreign DNA into mycobacteria. To achieve targeted gene knockouts, researchers also employed suicide vectors, such as pGOAL, pNIL, and pYUB, which carried DNA fragments with homologous regions flanking a selectable marker but lacked a functional origin of replication in the host, thereby promoting allelic exchange through a single recombination event[29-36](Table 2).

### Advances in Homologous Recombination: Specialized Vectors

Specialized vectors were designed to enhance the efficiency of homologous recombination by incorporating key features such as counter-selection markers (*e.g.*, *sacB*), site-specific integration systems, and two-step allelic exchange. Vectors incorporating counter-selectable markers (*e.g.*, new pGOAL and pPR27 series) were developed by expression in mycobacteria of structural *sacB* gene which encodes the *Bacillus subtilis* levansucrase (Table 2). The *sacB* expression is poisonous to mycobacteria in the presence of 5–10% sucrose [37]. Therefore, it was used to enrich for successful recombinants, gradually improving the efficiency of gene knockout experiments in NTMs [25] (Fig. 1A).

The pMV306 vector series were among the early integrating vectors that used the phage L5 integrase system for site-specific integration at the attB site in the mycobacterial genome [38] (Table 2). The integration system relies on two specific DNA sequences: the attP site (attachment site of the phage) present on the vector and the attB site (attachment site of the bacterium) found in the mycobacterial genome [39, 40]. These sequences are recognized by the phage L5 integrase enzyme, which binds both sites and aligns them to facilitate the integration process by creating a staggered cut in both attP and attB sites, then catalyzes the strand exchange, joining the vector and the bacterial chromosome precisely at these sites forming two new hybrid sites called attL and attR, which flank the integrated vector [41] (Fig. 1A). Integrated vectors can be unstable in mycobacteria, leading to plasmid loss. Researchers discovered that removing the integrase gene after integration makes the integrated vector much more stable within the mycobacterial genome [42]. When an integrase gene remains active in the cell, it can cause the replacement of one integrated vector by a second one if another L5-integrating vector is introduced. This effect can lead to instability of integrated vectors, as the integrase can facilitate the exchange of integrated vector. Removing the integrase gene after integration stabilizes the vector, preventing its exchange or excision and ensuring it remains in the genome [43].

Developed as allelic exchange vectors, p2NIL and pGOAL17, pMP1265 suicide vectors were designed to facilitate targeted gene replacements by applying two-step allelic exchange procedure to create unmarked mutants [22, 34, 44, 45] (Table 2). They combined specialized vectors with multiple resistance markers, negative selection marker (*sacB*), large homologous arms to the target gene and offering a two-step selection process: integration of the vector (single crossover) and then resolution (double crossover). The vector with homologous arms integrates into the bacterial chromosome via a single recombination event, selected by an antibiotic marker. The cointegrate is resolved through a second recombination event, replacing the target gene. Counter-selection with markers like *sacB* ensures only cells with successful allelic exchange survive, resulting in gene replacement or knockout. This two-step approach ensures high specificity and allows for precise gene replacements or knockouts, critical for studying gene functions in NTM. In addition, vectors such as pPR27, featured temperature-sensitive origins of replication, allowing researchers to control vector replication and integration conditions [46] (Table 2). At lower, permissive temperatures (*e.g.*, 30°C), pPR27 can replicate autonomously within mycobacterial cells. At higher, non-permissive temperatures (*e.g.*, 37°C or above), the plasmid cannot replicate independently. Under these conditions, cells lose the plasmid unless they integrate into the genome. The pPR27 vector, therefore, provides researchers with greater control, especially in species where traditional transformation methods are challenging.

### Recombineering: A Breakthrough for Mycobacterial Knockouts

The advent of recombineering vectors, like pJV53 or pNitET-SacB-kan, marked a significant advancement by incorporating phage-derived recombination proteins such as RecET or Che9c [22, 47] (Table 2). RecET is a pair of phage-derived proteins, RecE and RecT, that work together to mediate homologous recombination in bacterial cells. When the RecET proteins are expressed, they enhance the efficiency of homologous recombination by processing and pairing DNA for precise integration. The RecE protein creates single-stranded DNA by degrading one strand of a double-stranded break then RecT then binds to this ssDNA, stabilizing it and promoting its pairing with a homologous sequence in the target DNA, enabling strand invasion and exchange. These vectors increased the efficiency of homologous recombination, allowing for precise genetic modifications, including gene deletions and insertions. Recombineering greatly improved the versatility and success rates of homologous recombination in NTM.

Moreover, the introduction of Bxb1 integration system, a site-specific recombination system derived from the mycobacteriophage Bxb1, offers several advantages compared to the L5-integrating vector system [48, 49]. Bxb1 integrase is a serine recombinase, enabling highly efficient, site-specific integration with minimal sequence requirements for its attachment sites (attP and attB), often smaller than those required by L5 integrase. Additionally, Bxb1 is more precise, with less potential for recombination errors, and can operate in a broader range of contexts due to its smaller attachment sites without requiring supercoiled DNA [49]. Bxb1-mediated integration is typically more stable over time compared to L5 integration, which can be unstable if integrase is present in the system, not require counter-selection markers as the integration is specific and stable.

The ORBIT system (Oligonucleotide Recombineering followed by Bxb1 Integrase Targeting) reveals a new paradigm for genetic engineering of mycobacterial chromosomes [50]. This method combines recombineering (RecT recombinase of phage Che9c) and site-specific integration (Bxb1) to achieve precise genetic modifications, offering a more flexible and efficient alternative to traditional homologous recombination techniques (Fig. 1A). First, a *M. smegmatis* or *M. tuberculosis* strain is transformed with a plasmid that provides two key tools: RecT recombinase and Bxb1 integrase. Next, the bacteria are also transformed with a short synthetic DNA oligonucleotide and a separate plasmid that includes the Bxb1 attB site, an antibiotic resistance gene for selection, and the target DNA sequence. The oligonucleotide has a Bxb1 attP site surrounded by DNA regions that match the target chromosome, allowing for accurate insertion of the new DNA [22]. This dual approach enhances the precision and efficiency of gene knockouts, knock-ins, and other modifications, significantly advancing the genetic manipulation of *M. tuberculosis* and other NTM. The ORBIT system further allows the generation of conditional mutants, thereby expanding the toolkit for studying essential genes and pathways. Its ability to introduce changes without the limitations of homologous recombination makes it a breakthrough for mycobacterial genetic engineering. An example suicide vector, pKM444, a commonly used vector in the ORBIT system that facilitates gene knockouts in *M. smegmatis* and *M. tuberculosis*, features homology arms that flank the target gene, enabling homologous recombination for precise integration [50] (Table 2).

### Transposon Mutagenesis: Random Gene Knockouts

Transposon mutagenesis was first introduced in the late 1980s and early 1990s, providing a powerful tool to involve the insertion of transposons, or "jumping genes," throughout the genome, allowing researchers to disrupt multiple genes simultaneously and create large libraries of mutant strains [51]. This process starts with introducing a transposon, a mobile DNA element, into the bacterial genome. The transposon is carried on a plasmid and, once inside the cell, is integrated into the genome by the enzyme transposase, using a “cut-and-paste” mechanism where the transposon is excised from its original location and inserted into a new location in the genome. The insertion of the transposon typically disrupts the function of the gene at the integration site, resulting in a knockout of that gene (Fig. 1B). Researchers typically screen for mutants that show altered phenotypes, such as growth defects, resistance to antibiotics, or inability to survive under certain conditions. One of the earliest vectors successfully used for knockout genes using transposon mutagenesis in mycobacteria was pYUB285 carries the Tn5367 transposon [52-54] (Table 2). Tn5367 operates through a cut-and-paste mechanism, in which the transposon is excised from one genomic location and inserted into another. Although Tn5367 inserts largely at random, it displays a weak preference for AT-rich regions. This sequence bias may limit its utility in genomic contexts where more uniform or alternative insertion patterns are required. TnMod, Tn5-derived transposons vectors were designed to overcome some of the limitations of earlier systems, which allow random insertion throughout the mycobacterial genome with highly effective in generating mutant libraries in NTM like *M. abscessus* and *M. avium* [55]. Researchers can easily swap genetic elements within the transposon, such as selectable markers or regulatory elements, allowing for more customizable mutagenesis protocols. This flexibility enables the construction of various transposon variants to suit specific experimental needs. TnMod has been engineered to improve transposition efficiency, reducing the biases in insertion sites that are commonly seen with earlier systems. φMycoMarT7 vector system carry mariner transposons, specifically the Himar1 transposon, which randomly integrates into the genome [56, 57] (Table 2). Mariner transposons do not require specific target sequences for integration, making them ideal for generating random knockouts. Recently, deep sequencing of transposon (Tn) insertion libraries has emerged as a powerful method for assessing the essentiality of genomic elements in bacterial organisms. TnSeq (Transposon Sequencing) has revolutionized transposon mutagenesis by combining random transposon insertion with high-throughput sequencing. This approach generates a library of mutants with insertions mapped across the genome. By comparing mutant abundance under different conditions, researchers can determine which genes are essential or contribute to fitness. For example, genes disrupted in non-viable mutants are likely essential for survival, while others may which reveal roles in adaptation or pathogenicity [58]. Simplified, TnSeq involves mapping where transposons land in the genome and analyzing how their presence affects growth or survival under specific conditions. By introducing random Tn insertions, the functions of genes and regulatory regions can be disrupted. Despite its success, the random nature of transposon insertions meant that it was not suitable for targeted knockouts without additional screening steps.

### CRISPR-Cas9: Precision Knockouts

With the discovery of CRISPR-associated proteins (Cas proteins) and their role in recognizing and processing nucleotide sequences, CRISPR-Cas systems quickly evolved from a bacterial genetic curiosity to a groundbreaking tool for genetic modifications, revolutionizing the study of microbial physiology. The advent of CRISPR-Cas9 marked a new era in precision gene editing. Initially challenging to implement in NTM due to delivery and specificity issues, CRISPR-Cas9 has since been adapted to enable precise gene knockouts in various NTM species. This system utilizes a guide RNA (gRNA) to direct the Cas9 endonuclease to specific genomic loci, inducing double-strand breaks (DSBs). Once a DSB occurs, the cell can repair the breaks through two primary mechanisms: Non-Homologous End Joining (NHEJ) and Homology-Directed Repair (HDR) (Fig. 1C). NHEJ is an error-prone repair process that directly ligates the broken DNA ends, often resulting in insertions or deletions (indels) that can disrupt the target gene, effectively knocking it out. HDR, on the other hand, utilizes a homologous donor template to precisely repair the break, allowing for the introduction of specific mutations or the insertion of new sequences at the target site. Class II systems, including those from *Streptococcus pyogenes* (type II-A) and *Francisella novicida* (type V-A), use CRISPR RNA (crRNA) and Cas proteins to edit DNA [59]. The SpyCas9 endonuclease from *S. pyogenes* introduces double-strand breaks, guided by crRNA or single guide RNA (sgRNA). However, its toxicity in mycobacteria led to the development of a nuclease-deactivated version, dSpyCas9, and alternative Cas proteins like St1Cas9 from *Streptococcus thermophilus* [59-61]. St1Cas9 was effective with reduced toxicity when controlled by a regulated promoter (Table 2). The type V-A system from *F. novicida* also showed promise for genome editing but faced challenges with DNA repair due to double-strand breaks. New base editors allow precise nucleotide changes and are being adapted for mycobacteria. Class I type III-A CRISPR-Cas systems found in pathogenic mycobacteria can target both DNA and RNA. Recent advances include reprogramming these systems for genome editing and targeting mRNA in *M. tuberculosis*, providing tools for functional genomics and screening studies. The challenges include toxicity, particularly from SpyCas9, which can limit its use and requires controlled expression. Type V-A systems like FnoCas12a also present issues with DNA repair due to toxic double-strand breaks [22, 62]. The efficiency of these systems can be affected by the GC-rich nature of mycobacterial genomes and rare PAM sequences. Additionally, off-target mutations and promoter leakage can complicate results, highlighting the need for careful management of CRISPR-Cas applications. By facilitating targeted DNA cleavage and gene disruption, CRISPR-Cas9 has become a powerful and versatile tool for studying gene function in NTMs, allowing researchers to explore previously difficult questions.

### Conditional Knockout Systems

Condition knockout systems allow researchers to inactivate the gene only under specific conditions, such as the presence or absence of a particular inducer, such as in Tet-On/Tet-Off or auxotrophic system. A system for fast and slow-growing mycobacteria, including *M. tuberculosis*, using anhydrotetracycline (ATc) as an inducer was developed. These systems, based on the *E. coli* Tn10-derived tet regulatory system, feature a strong tetO-containing mycobacterial promoter, expression cassettes for the TetR repressor, and Atc. They enable gene regulation in *M. smegmatis* and *M. tuberculosis* with up to 20-fold below the minimal inhibitory concentration of ATc [63, 64]. The Tet-On/Tet-Off system offers precise control over gene expression, allowing reversible gene knockouts by switching gene activity on or off when necessary (Fig. 1D). Both systems use tetracycline (Tet) or its derivative doxycycline to regulate gene expression in a controlled and reversible manner. In the Tet-On/Tet-Off system, the tTA transactivator binds to a tetracycline-responsive element (TRE) in the gene promoter, activating transcription in the absence of tetracycline. Upon adding tetracycline, it binds to tTA, altering its structure and preventing it from binding to the TRE, thereby inhibiting gene expression. In the Tet-On system, the rtTA transactivator remains inactive without tetracycline. When tetracycline is added, it binds to rtTA, allowing it to bind to the TRE and activate gene transcription (Fig. 1D). However, the Tet-on/off system can exhibit leaky expression, meaning that even without the inducer, there may be some level of gene expression, which can complicate experiments. On the other hand, effective use of the system depends on the appropriate concentration of the inducer (*e.g.*, anhydrotetracycline), which must be optimized to avoid affecting bacterial growth or causing unintended effects with high potential toxicity.

### Bacteriophage-Based DNA Delivery

In response to the limitations of traditional DNA delivery methods, bacteriophage-based systems emerged as an effective means of introducing knockout constructs into NTM. Specialized transduction, a technique involving the use of mycobacteriophages to deliver DNA into bacterial cells, bypassed some of the barriers posed by the NTM cell wall [65]. The use of temperature-sensitive phages like phAE94 has increased transduction efficiency, enabling more successful genetic modifications in these bacteria [65-67] (Table 2). This approach has allowed more precise gene knockouts and has played a significant role in studies involving pathogenic NTM strains that were otherwise genetically intractable.

## Targeted Gene Suppression in Mycobacteria: Exploring Knockdown Methods

Knockdown techniques typically refer to methods that reduce the expression of specific genes rather than completely knocking them out, allowing scientists to study essential genes that cannot be completely deleted. Antisense RNA (asRNA) emerged as one of the earliest knockdown strategies [68]. In this method, small RNA molecules complementary to a target mRNA are expressed, leading to decreased translation of the gene. This approach was limited by poor efficiency, incomplete knockdown, and variability in different strains of mycobacteria. However, it was one of the first steps toward conditional gene suppression.

### TetR/pip OFF System for Inducible Knockdown

One of the first robust inducible knockdown systems developed for Mycobacteria was the TetR/pip OFF system, adapted from *E. coli* tetracycline-regulated systems [69, 70]. This system allows for the controlled expression of target genes using repressible promoter system effective in both fast- and slow-growing mycobacteria based on two chromosomally encoded repressors, dependent on tetracycline (TetR) and pristinamycin (Pip), respectively [70, 71]. This system provides a dual control mechanism: one repressor (TetR) regulates a second repressor (Pip). The target gene can only be expressed or repressed when both antibiotics (tetracycline/doxycycline and pristinamycin) are manipulated. While the TetR-Pip system offers excellent control for gene knockdown or expression, it can be limited by the complexity of its setup, potential leaky expression, the need for precise control of inducer concentrations, and potential physiological effects of the inducers. To enhance its stability and performance, researchers developed a new integrative vector with the integrase gene deleted, improving plasmid retention, and another vector carrying the Pip repressor gene to introduce a second chromosomal copy, reducing the risk of repressor loss or frameshift mutations [71-73]. Additionally, mutagenesis was employed to create a weaker Pip-repressible promoter, further improving the efficiency and performance of the TetR/Pip-OFF repressible system [73].

### CRISPR Interference (CRISPRi) for Gene Knockdown

**Fig. 2:**
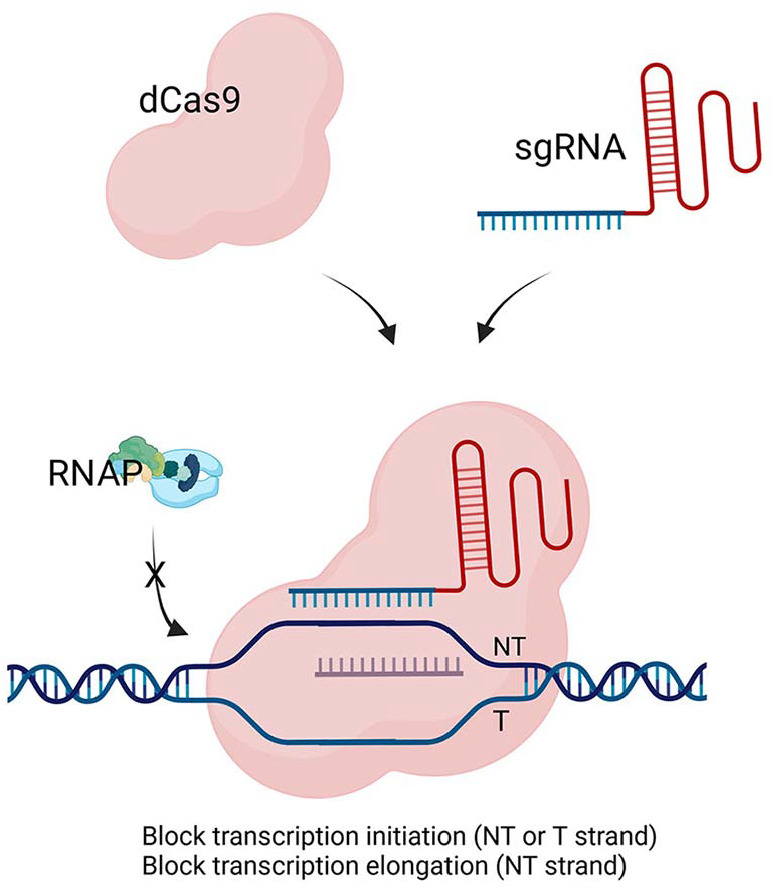
CRISPR interference (CRISPRi) using a catalytically inactive Cas9 (dCas9) that prevents transcription without cutting the DNA. By designing guide RNAs (gRNAs) that target either the non-template (NT) or template (T) strand of a gene, dCas9 can physically block RNA polymerase from accessing the promoter or coding region.

The advent of the CRISPR-Cas9 system revolutionized gene editing in both eukaryotes and prokaryotes. CRISPRi, a variation of CRISPR-Cas9, was adapted for mycobacteria to enable gene knockdown without causing double-stranded breaks [74, 75]. In CRISPRi, a catalytically inactive form of Cas9 (dCas9) is used, which binds to a target gene without cutting the DNA. When guided by a small RNA (sgRNA) to a specific region, dCas9 physically blocks transcription, leading to knockdown of gene expression [74, 75] (Fig. 2A). Depending on the delivery system, the plasmid may integrate into the genome or remain episomal. One of the most common ways to introduce CRISPRi components into Mycobacteria through plasmid is plasmid-based systems [76]. These plasmids typically carry the dCas9 gene under an inducible promoter and the sgRNA under a separate promoter [77]. Unlike gene knockout approaches that permanently disrupt gene function, CRISPRi allows for reversible and tunable knockdown controlled by varying the concentration of the inducer, allowing the study of essential gene function without killing the bacteria, avoids the introduction of double-stranded breaks in the DNA, preventing potential off-target effects and genomic instability. However, the efficiency of CRISPRi can vary depending on the sgRNA design and the accessibility of the target gene. Some sgRNAs may not completely block transcription, resulting in partial knockdown. Although CRISPRi is generally more specific than traditional knockdown methods, off-target binding of dCas9 can still occur, leading to unintended gene suppression. Effective sgRNA design is critical for CRISPRi efficiency and requires selecting a target sequence near a protospacer adjacent motif (PAM) site, such as the "NGG" sequence required by *S. pyogenes* dCas9 [78]. This PAM site must immediately follow the 20-nucleotide target sequence on the non-target strand for dCas9 binding and transcriptional repression. In mycobacteria, selecting sgRNAs near an "NGG" PAM site is essential for CRISPRi success. Factors like GC content (optimal range 40-60%), transcriptional accessibility, and the distance of the target site from the gene's transcription start site (preferably within 50-150 base pairs) are important for robust knockdown. Despite these design considerations, some sgRNAs may achieve only partial knockdown due to target accessibility. Validation of sgRNA specificity and knockdown efficiency is essential to minimize off-target effects and unintended gene suppression.

## Advances in Gene Overexpression for Mycobacteria: Tools and Limitations

Overexpression techniques in mycobacteria are used to artificially increase the expression of a specific gene, resulting in higher levels of the corresponding protein. This approach is essential for studying gene function, identifying potential drug targets, and understanding the role of certain genes in pathogenesis and metabolism. Overexpression can also be used to produce large amounts of proteins for biochemical and structural studies.

### Promoter Systems for Overexpression

The overexpression system consists of a strong promoter for driving high levels of gene expression (*hsp60*, *ptet*, or *Pmyc1* promoter or the *groEL* promoter). In addition to constitutive promoters such as *hsp60* and *Pmyc1*, recent studies have developed controllable promoter systems to achieve tunable gene expression in *Mycobacterium* species. One representative example is the work by Boldrin *et al*., who designed a dual repressor-based promoter system using TetR and Pip regulators [73]. In that study, several promoters including *Pptr*, *P606*, *Psmyc*, and *Phsp60*, were comparatively analyzed to generate a spectrum of expression strengths [73]. This approach demonstrated that transcriptional output can be precisely adjusted through promoter selection and repressor control, expanding the available tools for modulating gene expression in mycobacteria for both overexpression and conditional regulation purposes [73]. Episomal vectors, like the pMV261 series, enable robust gene expression without altering the bacterial genome, making them ideal for transient studies. Several studies have successfully applied this vector to overexpress genes in *M. tuberculosis* and *M. smegmatis*. For example, the pMV261 vector has been used to overexpress *Mtb* Rv0927, a transcriptional regulator involved in stress response [79], and *Rv2629* involved in the survival of the clinical drug-resistant strain via bacterial growth repression and bacterial persistence induction [80]. These systems have enabled functional studies of genes in mycobacteria, providing valuable insights into their roles in virulence, survival, and stress adaptation. Several vectors based on the pMV261 backbone have been engineered by replacing its *hsp60* promoter with acetamide- or tetracycline-inducible promoters. These vectors also allow fusion of affinity or reporter tags—such as hexahistidine, GFP, FLAG, or GST—at either the N- or C-terminus of the expressed protein [81]. The *Mycobacterium*–*E. coli* shuttle vector system based on the pAL5000 replicon has long been used for heterologous gene expression in mycobacteria, although its utility is limited by relatively low copy number and expression efficiency. More recently, the linear plasmid pMyong2 from *M. yongonense* has been introduced as a compatible alternative, exhibiting ~37-fold higher copy number and markedly enhanced protein expression, thereby providing a promising complement to conventional pAL5000-based vectors [82].

In contrast, integrative systems like pMV306 integrate the overexpression construct into the chromosome, offering stable, long-term expression, which is crucial for studying essential genes or those that could be toxic at high levels. This system has been used to study genes from *M. neoaurum* [83], *M. smegmatis* [84]. Integrative systems are especially valuable for studying essential or toxic genes, as they allow for the sustained, controlled expression necessary for in-depth functional analysis without compromising bacterial survival.

Recently, systematic strategies have been developed to expand the repertoire of promoters available for mycobacterial expression systems. The mutagenized promoter library derived from the strong P_L5_ promoter of mycobacteriophage L5, generating variants with high (pJK-F8), intermediate (pJK-B7, pJK-E6, pJK-D6), and low (pJK-C1) strengths were used in both *M. smegmatis* and *M. bovis* BCG [85]. This promoter library was further validated by driving expression of the *Schistosoma mansoni* antigen Sm29 in BCG, illustrating its potential for vaccine development, synthetic biology, and systems biology studies requiring precise control of gene expression.

## Emerging Tools and Future Directions

While classical methodologies, such as homologous recombination and CRISPR interference, have established the groundwork for mycobacterial mutagenesis, several innovative techniques are emerging in other model systems that hold potential for future applications. CRISPR activation (CRISPRa) platforms facilitate the upregulation of targeted endogenous genes through the recruitment of transcription factors, which may be particularly beneficial for elucidating the regulatory networks of *M. tuberculosis* and NTM [86]. Additionally, prime editing, a next-generation technology that combines Cas9 nickase with reverse transcriptase, provides precise single-nucleotide substitutions without generating double-strand breaks, which is advantageous in GC-rich genomes that are prone to repair-associated toxicity [87, 88]. Beyond DNA-based editing, automated genetic circuit design and synthetic biology approaches are increasingly being explored in various bacteria, including *E. coli* and *B. subtilis* [89-91] and could eventually be adapted to mycobacteria for programmable control of metabolic or virulence pathways [92, 93]. Furthermore, cell-free systems provide a powerful complementary strategy, allowing the rapid prototyping of genetic constructs and protein expression without the constraints of the mycobacterial cell wall and slow growth [94, 95]. Although these technologies remain largely prospective for mycobacteria, their integration into the mycobacterial genetic toolbox could markedly accelerate drug discovery and therapeutic innovations.

## Comparative Analysis of Available Tools

Each genetic engineering strategy in mycobacteria has distinct advantages and limitations. The recombineering and ORBIT systems resemble a surgical scalpel, offering high precision with specialized recombinases. However, these methods require complex technical setups, making them less accessible in resource-limited laboratories [50, 96]. In contrast, transposon mutagenesis resembles a shotgun approach, being highly applicable for genome-wide functional screening, enabling systematic analysis of gene function [97, 98]. Its major limitation is the lack of specificity, necessitating the use of extensive mutant libraries and subsequent, time-consuming mapping procedures [99].

CRISPR-based methods provide unparalleled flexibility and precision in targeted genetic modifications. However, their application faces several significant constraints: Cas9 toxicity in mycobacterial cells, limited availability of PAM sequences in GC-rich mycobacterial genomes, and the requirement for stringent promoter regulation to control nuclease expression [99]. Notably, studies have developed engineered Cas9 variants with reduced toxicity and modified PAM requirements to overcome some of these challenges [74, 75, 100]. For instance, the use of dCas9 systems, which are catalytically inactive, can sidestep toxicity issues while still providing robust gene expression control [75]. Publications have demonstrated the effectiveness of these altered systems in GC-rich environments, showcasing a practical workaround to the PAM scarcity dilemma [74, 75, 100].

Conditional expression systems, such as Tet-On/Tet-Off, are invaluable for investigating essential genes whose knockout would be lethal [63, 101, 102]. Nevertheless, leaky expression in the "off " state and potential inducer toxicity can complicate data interpretation, affecting cell viability. For overexpression studies, episomal vectors offer a straightforward and robust approach that allows rapid protein production for biochemical analyses [103, 104]. However, they may impose a metabolic burden on host cells and show copy number variations. In contrast, integrative systems provide superior genetic stability by incorporating constructs into the chromosome, but this comes at the expense of flexibility and requires more time-consuming strain constructions [105, 106].

**Fig. 3:**
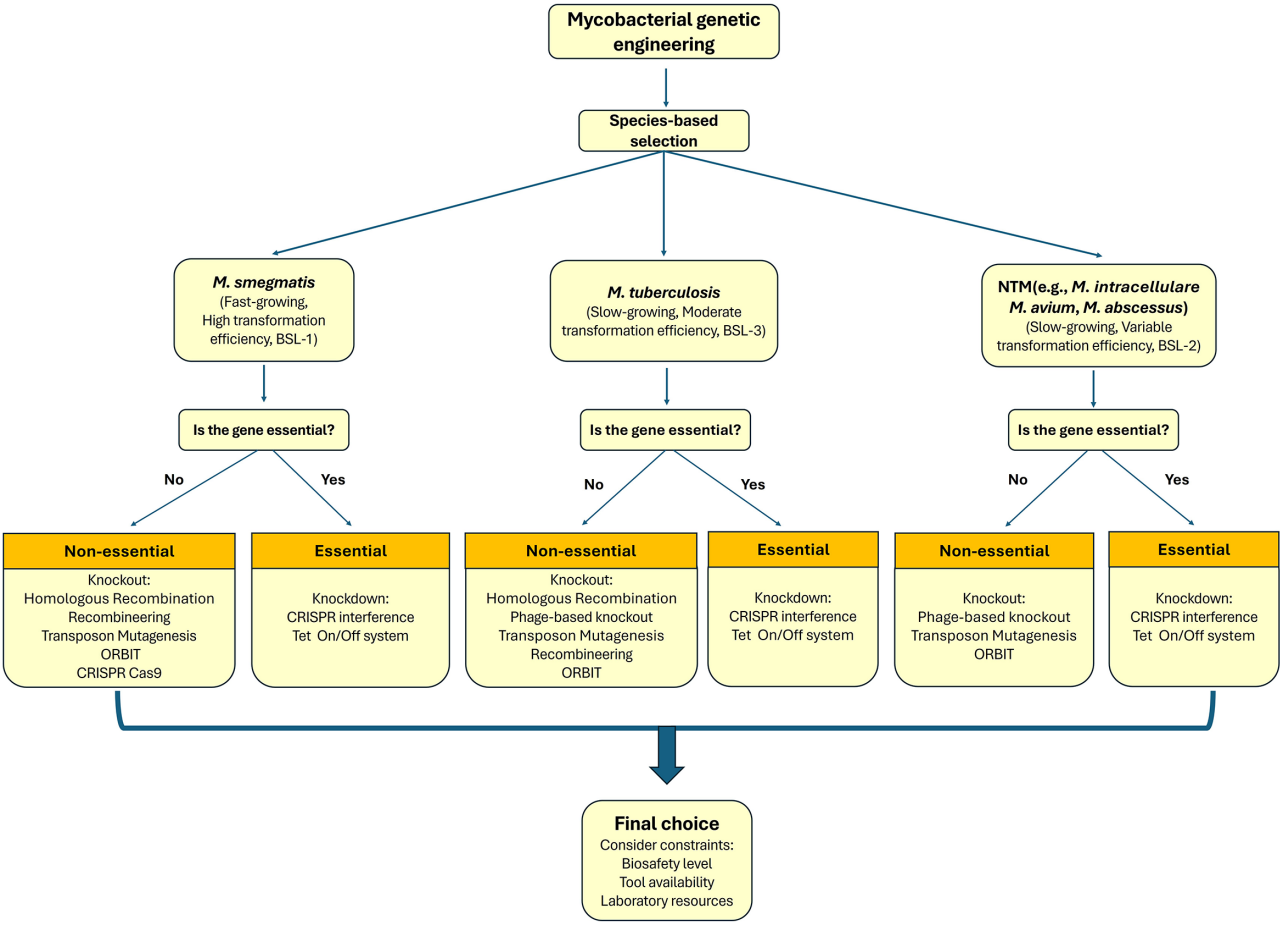
Decision tree for selecting genetic engineering strategies in mycobacteria. The decision process begins with the choice of mycobacterial species, which strongly influences tool availability. For example, *M. smegmatis* is amenable to homologous recombination and high-throughput mutagenesis; *M. tuberculosis* requires BSL-3 facilities and benefits from specialized phage transduction; and non-tuberculous mycobacteria (NTM) often pose transformation efficiency challenges. Within each species, gene essentiality determines whether knockout (non-essential) or knockdown/conditional expression (essential) approaches are appropriate. Manipulation type (knockout and knockdown) is then matched to specific tools (*e.g.*, recombineering, CRISPRi, ORBIT). Finally, experimental constraints such as biosafety level and resource availability guide the selection of the most feasible system.

To effectively select genetic tools for mycobacterial engineering, consider using a decision tree approach (Fig. 3):

1. Select species: Start by selecting mycobacterial species that strongly affect tool availability.

2. Evaluate gene essentiality: If the gene of interest is essential, lean towards dCas9 or Tet-On/Off systems; however, be mindful of potential inducer toxicity and leaky expression.

3. Consider Biosafety Regulations and infrastructure: Ensure that your method aligns with local regulatory requirements. In addition, evaluate your laboratory’s capacity. If resources are limited, avoid methods requiring complex setups, such as recombineering, and consider more straightforward options, such as transposon mutagenesis.

By following these steps, the approach can be tailored based on specific research needs and constraints. Understanding the strengths and limitations of each method is critical for successful mycobacterial genetic engineering.

## Conclusion

The genetic engineering of mycobacteria remains a complex but essential effort in mycobacterial disease research. Despite the significant challenges of slow growth rates and impenetrable cell walls, innovations such as recombineering, ORBIT-mediated site-specific gene editing, and CRISPR platforms have dramatically enhanced genetic manipulation capabilities. Knockout strategies continue to evolve through refined homologous recombination and transposon mutagenesis techniques, while conditional knockdown systems and CRISPR interference provide powerful approaches for studying essential genes. Overexpression systems contribute to functional analysis and protein production but require careful vector and promoter selection. No single method is universally optimal, and tool selection should be tailored to the specific goals of each experiment. By systematically reviewing and comparing available genetic approaches, this study provides a valuable framework to guide future research into the molecular pathogenesis and treatment of mycobacterial infections across species.

## Figures and Tables

**Table 1:** Summary of Gene Knockout Methods in mycobacteria.

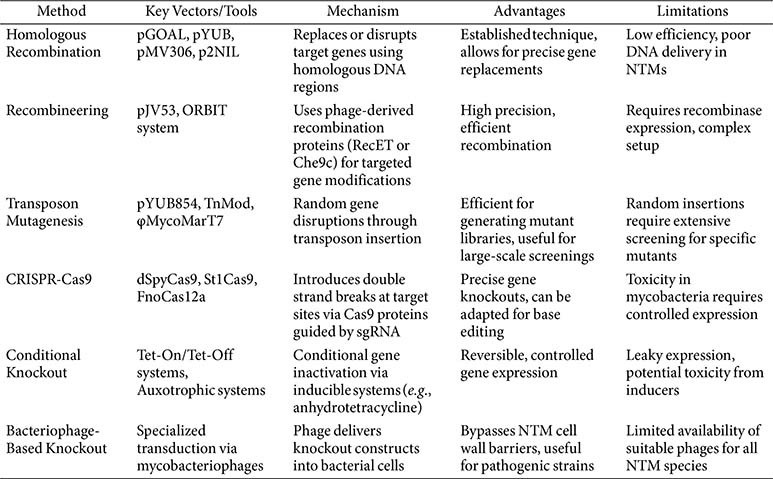

**Table 2:** Characteristics of Key Vectors for Gene Knockout in mycobacteria.